# Supplementary material for: A benchmarking study of feature screening approaches across type 1 diabetes omics studies classification settings
Source: PLoS Comput Biol. 2026 Aug 12;22(8):e1014625. doi: 10.1371/journal.pcbi.1014625 (PMC13465820; doi:10.1371/journal.pcbi.1014625)
Supplement: Supplementary file 1 [file pcbi.1014625.s001.docx]

# Supplementary Information

**Supplementary Fig 1. Simulated Example AUC:**  The AUC for feature recovery in the simulated data example is displayed as a function of sample size.
